# Prospective screening for sexually transmitted infections among US service members with *Chlamydia trachomatis* or *Neisseria gonorrhoeae* infection

**DOI:** 10.1371/journal.pone.0280783

**Published:** 2023-01-20

**Authors:** Sheryl Bedno, Shilpa Hakre, Shannon Clark, Nicole Dear, Mark Milazzo, Amy McCoart, Zebiba Hassen, Heather Liu, Elizabeth J. Bianchi, Janice M. Darden, Misti Paudel, Jennifer A. Malia, Sheila A. Peel, Paul T. Scott, Bruno Petruccelli

**Affiliations:** 1 Rutgers New Jersey Medical School, Newark, New Jersey, United States of America; 2 Emerging Infectious Diseases Branch, Walter Reed Army Institute of Research, Silver Spring, Maryland, United States of America; 3 Henry M. Jackson Foundation for the Advancement of Military Medicine, Inc., Bethesda, Maryland, United States of America; 4 US Military HIV Research Program, Walter Reed Army Institute of Research, Silver Spring, Maryland, United States of America; 5 Diagnostics and Countermeasures Branch, Walter Reed Army Institute of Research, Silver Spring, Maryland, United States of America; GGD Amsterdam, NETHERLANDS

## Abstract

**Background:**

*Chlamydia trachomatis* (CT) and *Neisseria gonorrhoeae* (NG) are the most common bacterial causes of sexually transmitted infection (STI) in the United States (US). The purpose of this study was to determine the frequency of reinfection during a six-month study period and to evaluate the retesting interval for those infected with CT or NG.

**Methods:**

We conducted a prospective, six-month follow-up study among US military personnel with new onset, laboratory-confirmed CT or NG, recruited from an STI clinic at a large military base from January 2018 to January 2020. Each participant was randomly assigned to one of four groups, which differed only by the timing of the first study-associated follow-up visit after CT or NG diagnosis.

**Results:**

Of the 347 initially recruited into the study, 267 participants completed a follow-up visit prior to their scheduled, final visit 6 months after initial infection. The median age at enrollment was 22 years and 41.0% were female. There were 32 (12.0%) reinfections (30 CT and 2 NG) after treatment of an index diagnosis of CT or NG within the six-month study period. Six of the CT reinfections were only detected at the final visit. A review of medical records revealed additional CT and NG reinfections. The probability of detecting a reinfection did not vary significantly by timing of follow-up.

**Conclusions:**

The likelihood of detecting CT or NG reinfection did not differ according to time of follow up visit among study participants, thus supporting CDC guidance to retest three months post treatment. Efforts should continue to focus on STI prevention and risk reduction.

## Introduction

In 2020, 1.6 million *Chlamydia trachomatis* (CT) infections and more than 677,000 *Neisseria gonorrhoeae* (NG) infections were reported in the United States (US), making these the most common bacterial causes of sexually transmitted infection (STI) in the US [1]. The incidence rates of reported CT and NG infections increased steadily between 2000 and 2019, in both the US population as a whole and the US military population specifically [1, 2]. A study of 2015–2019 chlamydia and gonorrhea rates among US Army soldiers, compared to the general US population, showed higher age- and sex-adjusted rates of chlamydia, but lower gonorrhea rates [3]. US rates are based on disease notifications, and estimated using the total US population, whereas the rates for the US military are based on Department of Defense (DoD) health surveillance data (both notifications and electronic health records) and are calculated as incidence densities, so actual rate differences may not be as high as we observed [4]. Furthermore, there is universal access to healthcare by active duty service members, including regular screening as part of wellness care for women, which facilitates detection of asymptomatic chlamydia cases.

Regardless of military-civilian comparisons, CT and NG infections are of particular importance to the DoD due to their negative impact upon military readiness. STIs are now among the most amenable to mitigation due to the availability of effective treatments and preventive interventions. Complications due to chlamydial and gonococcal infections not only consume significant healthcare costs but adversely affect the retention and deployable status of soldiers [2, 5].

The US Centers for Disease Control and Prevention (CDC) recommend retesting at 3 months post-treatment for both CT and NG [6], and the International Union Against Sexually Transmitted Infections cites the European guideline that recommends retesting at 3–6 months for chlamydia [7] and a test of cure at 3–7 days after completion of therapy for gonorrhea with consideration of repeat testing a week later [8, 9]. Retesting of patients following treatment for CT or NG infection has been an important component of disease control for many years since clinical interventions to prevent CT and NG are limited. Since nucleic acid amplification testing (NAAT) became the standard for detecting CT and NG, national guidelines (and international guidelines for chlamydia) have included a caution against using a ‘test of cure’ at 3 to 4 weeks post-treatment in cases of genital infection, except in pregnant patients [6, 7]. Delaying repeat tests beyond a month minimizes the possibility of falsely attributing the persistence of genetic material to viable bacteria. Further supporting this advice has been the observation that reinfection, and not treatment failure, accounts for most of the positive results discovered on follow-up testing [6]. Thus, the emphasis during the early days and weeks following infection should be on educating patients and ensuring treatment of sex partners.

Reinfection remains not only an important problem globally, but among military service members. In an analysis of reportable disease data conducted prior to the present study, we found that the recurrence of CT infection among Fort Bragg soldiers who were treated for a prior CT infection was 10.6% within a median period of 5.8 months [10]. While this observation is similar to studies of non-military populations, it is relatively high considering the active follow-up, case contact tracing, and counseling procedures practiced at Fort Bragg, which has a specialized, readily accessible, and discretely operated STI clinic. Furthermore, the actual incidence rate of recurrent infection was 110.7 per 1000 person years (11.1 per 100 person years); double the observed recurrence rate previously observed from 1994–1999 across the Army as a whole [11].

The purpose of this study was to evaluate the frequency of reinfection within a six-month period and validate, to the degree possible, the three-month retesting interval in the US military population, which is subject to relatively unique community transmission patterns. Service members come from locations throughout the nation and its territories; and most, whether in garrison or deployed, are in a setting that would not be considered urban, in contrast to pertinent STI follow-up studies since 2000. In addition, since military healthcare providers place a high priority on readiness and often resort to a test of cure, in addition to national guidelines and clinical judgement, as the evidence basis for additional treatment—particularly on bases where local STI rates are high—this study was designed to inform same. A study characterizing the dynamics of disease recurrence on a military base where the occurrence of STIs is a persistent public health problem can provide evidence to inform revision of current practices within the Military Health System. Furthermore, preventing reinfection more effectively could have a multiplicative, beneficial effect on the health of both active duty service members and civilian populations in communities surrounding military installations.

## Method

### Study design

This was a prospective, follow-up study of CT- and NG-infected patients at Fort Bragg, North Carolina (NC). The Fort Bragg Department of Public Health operates a dedicated STI clinic with effective diagnosis, treatment, and follow-up procedures in place.

### Ethical considerations

This study adhered to the ethical guidelines from the Walter Reed Army Institute of Research and Womack Army Medical Center. Institutional Review Board approval was provided by the Walter Reed Army Institute of Research (WRAIR # 2236). All participants gave their consent to participate by signing a printed form that described the purpose, procedure, risks and benefits of the study.

### Study population

CT and NG patients seeking care during the period January 2018 to January 2020 were invited to participate. Male and female US Army soldiers who were at least 18 years of age and receiving care at the Epidemiology and Disease Control clinic at Fort Bragg, were eligible if they had new onset CT or NG infection confirmed by nucleic acid amplification testing (NAAT) of a urine or genital specimen in the previous 60 days, regardless of the antimicrobial treatment received. In addition, participants had to be permanently assigned to Fort Bragg, be expected to remain at the base for at least 6 months beyond study entry, and be willing to provide appropriate specimens (urine from men, vaginal swabs or urine from women, and blood specimens from both men and women). Uncomplicated pregnancy did not exclude participation.

Each volunteer was randomly assigned to 1 of 4 groups, which differed only by the timing of the first study-associated follow-up visit: 2, 3, 4, or 5 months from CT or NG diagnosis. Study-related follow-up visits were separate from a test-of-cure visit at 21 to 28 days after treatment required under local STI clinic procedures. All participants were also seen at 6 months after diagnosis. Participants were permitted to change their follow-up appointment so that it occurred up to 15 days before or after the scheduled date and were allowed up to 60 days beyond the scheduled, 6-month visit to comply with the final encounter. Participants were compensated US $50 for blood collection at each follow-up visit.

Outcomes recorded during the follow-up period included any newly detected case of an STI (including CT or NG reinfection). A questionnaire administered at enrollment served as the source of demographic data. The local, permanently assigned, clinical and laboratory staff of the Womack Army Medical Center provided all services normally prompted by a case of CT or NG, such as counseling, contact tracing and antimicrobial treatment associated with both the initial and any subsequently diagnosed infection.

### Laboratory testing

Study-directed testing for the following agents was conducted at each scheduled encounter using US Food and Drug Administration-cleared assays: CT, NG, *Trichomonas vaginalis* (TV), *Treponema pallidum* (TP), hepatitis B virus (HBV), hepatitis C virus (HCV), and human immunodeficiency virus (HIV). Local laboratory testing for additional agents was performed, if clinically indicated. The following specimens were obtained at each study encounter. A first-catch urine specimen from men and vaginal swab or urine specimen from women were collected for CT, NG, and TV testing by NAAT. Self-collection was permitted for vaginal swabs. Specimens were transferred to transport media specific for urine and vaginal secretions, respectively. Blood (20 milliliters) for serologic analysis was obtained by venipuncture. Genitourinary and centrifuged blood specimens were shipped to the HIV Diagnostics & Reference Laboratory, Diagnostics and Countermeasures Branch, Walter Reed Army Institute of Research Silver Spring, Maryland, where all testing was conducted. Nucleic acid amplification testing using the Aptima Combo 2^®^ assay was utilized to detect CT, NG, and the Aptima Trichomonas Vaginalis assay to detect TV (Hologic Canada ULC) in genitourinary specimens. Serologic assays conducted were the following: Wampole Impact Rapid Plasma Reagin (Abbott Laboratories, Abbott Park Illinois, US) for *T*. *pallidum*, GS HBsAg Confirmatory Assay 3.0 (BioRad Laboratories, Hercules, California (CA), US) for confirmation of hepatitis B surface antigen, GS HIV Combo Ag/Ab EIA for detection of HIV-1 /HIV-2 (BioRad Laboratories, Redmond, Washington (WA), US), and Ortho^®^ HCV Version 3.0 ELISA (Chrion Corporation, Emeryville, CA, US). Any positive Rapid Plasma Reagin, HCV antibody or HIV antigen/antibody test prompted reflex testing by *T*. *pallidum* particle agglutination (Quest Diagnostics, Chantilly, Virginia, US), COBAS®AmpliPrep/COBAS® TaqMan® HCV Test v2.0 (Roche Diagnostics, Indianapolis, Indiana, US) for detection of HCV RNA, and a GS HIV-1 Western Blot for HIV-1 and the Geenius HIV ½ Supplemental Assay for HIV-1/2 (BioRad Laboratories, Redmond, WA, US).

### Medical record review

Electronic health records were searched for results of any CT, NG, TV, syphilis, HBV, HCV, and HIV tests ordered for participants. Test dates, results, and treating facility were extracted for inclusion in a sensitivity analysis.

### Statistical analysis

The required sample size was calculated based on estimated, 6-month reinfection rates determined from DoD and Fort Bragg-specific health surveillance data, assuming an alpha level of 0.05 and a power (1—beta) of 80%. Statistical tests such as Pearson’s chi-square, Kruskal-Wallis, Fisher’s exact tests were used to compare demographic characteristics at enrollment and proportion of reinfections by group. Reinfection was defined as a first CT or NG infection detected more than 6 weeks (42 days) after the date of the positive CT or NG NAAT that determined eligibility for the study (index date). Exceeding the 30-day window [12] avoided misclassification of post-treatment specimens that effectively served as tests of cure. An overall reinfection rate among study participants was determined, for which individual person-time at risk was considered from 6 weeks after the index date through the date of the last study encounter.

An intention-to-treat approach was applied in the main analysis, such that participants were retained in the group to which they were allocated, even if their first follow-up visit occurred outside the allowable window of the assigned encounter date. A separate analysis examined whether or not results would differ substantially if they were based on actual times to reinfection. In this approach the original group allocation was disregarded, and participants were reclassified to the follow-up interval group determined by the first date when they actually had a follow-up encounter with testing performed. This analysis also included any positive test results, determined by medical record review, that were not generated by the study. Again, visits and tests within 6 weeks of the index date were excluded. Dual entry of questionnaire and chart review data was performed in Research Electronic Data Capture (REDCap). Statistical analyses were performed in SAS version 9.4 (SAS Institute, Cary, NC) and Stata version 16.1 (StataCorp, College Station, Texas) software.

## Results

A total of 347 participants were initially enrolled in the study. Of these, 267 completed a first follow-up visit, and 224 completed both a first follow-up visit and a final (6-month) visit. In addition, there were 10 participants who were only able to comply with the 6-month visit. Table 1 shows the distribution of participants by their assigned follow-up time interval; Table 2 shows participant demographic characteristics. The median age was 22 years (interquartile range (IQR 20–25 years); 41% (n = 110) were female. Out of 312 specimens collected from women, 302 (97%) were by vaginal swab and 10 (3%) were urines. The only significant difference among groups was the higher proportion of Hispanic participants in the 2- and 3-month groups compared to the 4- and 5-month groups.

**Table 1:** Description of participation by follow-up visit and allocation group.

Assigned follow-up interval	Number allocated	Completed a first follow-up visit	Completed both follow-up and final (6-month) visits
2 months	77	64	48
3 months	86	69	58
4 months	83	64	56
5 months	101	70	62
Totals	347	267	224

Ten participants who only complied with the 6-month visit are excluded from the table.

**Table 2:** Demographic characteristics of study participants who completed a follow-up visit prior to the final (6-month) visit.

Assigned follow-up interval->	2 months	3 months	4 months	5 months	Total	Overall p-value
	n = 64	n = 69	n = 64	n = 70	n = 267	
**Age (years), median (IQR)**	21.5 (20–23)	22 (20–26)	23 (20–25)	22 (20–24)	22 (20–25)	0.36
**Age (years), mean (SD)**	22.2 (3.2)	23.0 (4.1)	23.2 (3.5)	22.4 (2.6)	22.7 (3.4)	0.22
**Age Group (years)**						0.09
18–20	20 (31.3%)	23 (33.3%)	18 (28.1%)	21 (30.0%)	82 (30.7%)	
21–22	21 (32.8%)	14 (20.3%)	10 (15.6%)	20 (28.6%)	65 (24.3%)	
23–25	16 (25.0%)	14 (20.3%)	24 (37.5%)	21 (30.0%)	75 (28.1%)	
26–38	7 (10.9%)	18 (26.1%)	12 (18.8%)	8 (11.4%)	45 (16.9%)	
**Sex**						0.19
Male	35 (54.7%)	42 (60.9%)	44 (68.8%)	36 (51.4%)	157 (58.8%)	
Female	29 (45.3%)	27 (39.1%)	20 (31.3%)	34 (48.6%)	110 (41.2%)	
**Ethnicity**						0.002
Hispanic	21 (32.8%)	20 (29.0%)	8 (12.5%)	8 (11.4%)	57 (21.3%)	
Non-Hispanic	39 (60.9%)	46 (66.7%)	52 (81.3%)	61 (87.1%)	198 (74.2%)	
Unknown	4 (6.3%)	1 (1.4%)	2 (3.1%)	1 (1.4%)	8 (3.0%)	
Missing^a^	0 (0.0%)	2 (2.9%)	2 (3.1%)	0 (0.0%)	4 (1.5%)	
**Marital status**						0.13
Single, never married/not in partnership	25 (39.1%)	29 (42.0%)	20 (31.3%)	21 (30.0%)	95 (35.6%)	
Not married but in committed relationship	21 (32.8%)	14 (20.3%)	15 (23.4%)	21 (30.0%)	71 (26.6%)	
Married	12 (18.8%)	15 (21.7%)	10 (15.6%)	17 (24.3%)	54 (20.2%)	
Separated/divorced	6 (9.4%)	11 (15.9%)	19 (29.7%)	11 (15.7%)	47 (17.6%)	
**Education**						0.15
HS Diploma	34 (53.1%)	36 (52.2%)	36 (56.3%)	31 (44.3%)	137 (51.3%)	
Some college	21 (32.8%)	23 (33.3%)	22 (34.4%)	32 (45.7%)	98 (36.7%)	
Vocational/technical school or associate degree	2 (3.1%)	5 (7.2%)	6 (9.4%)	4 (5.7%)	17 (6.4%)	
Bachelor’s or graduate degree	7 (10.9%)	5 (7.2%)	0 (0.0%)	3 (4.3%)	15 (5.6%)	
**Current pay grade** ^a^						0.22
E1-E3	23 (35.9%)	31 (44.9%)	20 (31.3%)	29 (41.4%)	103 (38.6%)	
E4-E8	37 (57.8%)	37 (53.6%)	43 (67.2%)	41 (58.6%)	158 (59.2%)	
O1-O3	3 (4.7%)	1 (1.4%)	0 (0.0%)	0 (0.0%)	4 (1.5%)	
Missing ^b^	1 (1.6%)	0 (0.0%)	1 (1.6%)	0 (0.0%)	2 (0.7%)	

Categorical data are presented as n (column %), and continuous data are presented as median (IQR). Pearson’s chi-square, Fisher’s exact test, and Kruskal-Wallis (for non-normally distributed variables) tests were used to compare across groups.

^a^ Approximate base salary ranges by pay grade and years of service can be found at https://www.goarmy.com/benefits/while-you-serve/money-pay.html; for example a soldier with a pay grade of E1 and O3 earn a starting salary of $21,999.60 per year and $55,638.00, respectively.

^b^ This category was not included as a separate level in models.

The intention-to-treat analysis revealed a total of 30 CT and 2 NG reinfections, which translate to incidence rates per 100 person-years of 30.6 (95% CI 21.4–43.8) and 2.0 (95% CI 0.5–7.9), respectively. Twenty-four of the CT reinfections and both of the NG reinfections were detected at the first follow-up visit. No participants were observed to develop a coinfection with CT and NG following their initial STI diagnosis. The probability of detecting a reinfection did not vary significantly by the length of the follow-up interval (p = 0.72, Table 3).

**Table 3:** Incidence of reinfection with *Chlamydia trachomatis* or *Neisseria gonorrhoeae*.

Assigned follow-up interval	At first follow-up ^a^			
	Total N	N (%) of CT reinfections	N (%) of NG reinfections	N (%) of participants with CT or NG reinfection ^b^
2 months	64	3 (4.7)	1 (1.6)	4 (6.3)
3 months	69	8 (11.6)	0 (0)	8 (11.6)
4 months	64	6 (9.4)	1 (1.6)	7 (10.9)
5 months	70	7 (10.0)	0 (0)	7 (10.0)
All groups	267	24 (9.0)	2 (0.7)	26 (9.7)

Abbreviations: CT = *Chlamydia trachomatis*; NG = *Neisseria gonorrhoeae*. Data are presented as n (row %).

^**a**^ If an individual tested positive at both follow-up visits, only the first positive result was considered.

^**b**^ p-value = 0.72 (Fisher’s exact test was used to compare the number of participants with CT or NG reinfection by group at the first follow-up visit)

For the sensitivity analysis, participants were reassigned to the follow-up month (2, 3, 4, or 5) within 2 weeks of their actual, first encounter beyond six weeks from the index date, regardless of the month to which they were originally allocated. The proportion of persons with CT or NG reinfection did not vary significantly by the number of months from diagnosis of the original infection. As in the intention-to-treat analysis, no demographic characteristics were associated with an increased risk of reinfection.

Six new cases of *T*. *vaginalis* were diagnosed during the study period (incidence rate 7 per 100 person-years) in addition to 4 cases that had been detected at the time of enrollment. No incident cases of *T*. *pallidum*, HBV, HCV, or HIV infection were detected throughout the study period.

## Discussion

This is the first study conducted in a US military population to evaluate different STI re-testing intervals. The findings indicate that the timing of a scheduled follow-up visit after diagnosis of initial CT or NG infection had no impact on the likelihood of detection of reinfection between 2 and 6 months after the first positive test event. Furthermore, in this relatively limited cohort of active duty US military personnel with an initial CT infection, the likelihood of reinfection by either agent could not have been predicted based on age, sex, marital status, paygrade, or education level.

There have been few studies in non-military populations randomizing to different STI retesting time periods. One larger study (n = 2,253), based in an Amsterdam STI clinic, re-tested participants for CT at 2, 4, or 6 months and found a higher retesting uptake in the 2 month group [13]. The study did not include those with NG, nor did it evaluate the 3-month retesting interval, acknowledging that 3 months is a common testing interval in several guidelines. The study authors recommended retesting at 2 months due to the higher retesting uptake and comparable positivity proportion versus 4- and 6-month groups.

Several other relevant studies have evaluated retesting among those diagnosed with CT and/or NG. In a cluster randomized study of over 2,000 individuals in Australia diagnosed with CT or NG, Garton et al. evaluated the proportion of those retested at <2 months (“not recommended”), 2–4 months, and 5–12 months, plus those with repeat positivity [14]. Approximately 15% were retested at 2 months and 27% at 5–12 months. This study, compared to ours and the study in Amsterdam, focused on retesting compliance versus randomizing prospectively to different timelines. In a study of 1,165 individuals having NG with reduced antimicrobial susceptibility, 61% of individuals returned for a test of cure [15]. The study reported that it did not detect any treatment failures; and moreover, that positive test events were likely due to reinfection or false positive NAAT results. Although the CDC does not recommend a test of cure in most cases, it does recommend it for pregnant women, pharyngeal NG, and suspected cephalosporin treatment failure among those treated for NG [6].

This study had several limitations. Although participants were informed of the need to remain in the study for six months, many participants did not complete follow up visits. Military personnel may have had unexpected training, military mission requirements, or deployments; therefore, were not available for study participation. The results of this study may not be generalizable to other populations because of the uniqueness of military populations: military personnel are generally healthy, have access to universal, free health care, and are screened regularly for HIV and other STIs. Furthermore, the study participants were recruited from a dedicated STI clinic and findings may not be generalizable to a lower risk population. Also, while we were able to successfully meet recruitment goals and gather robust data, the coronavirus disease 2019 (COVID-19) pandemic prevented completion of final follow-up visits for some participants. Finally, despite STI clinic procedures that ensure antimicrobial treatment of NAAT-positive patients, and retreatment of those still positive at their test-of-cure appointment, misclassifying a persistent infection as reinfection was not ruled out by genetic analysis of the pathogens.

## Conclusions

This study sought to evaluate the optimal CT or NG retesting interval in the US. military population. The question of when to retest is particularly important for CT infection, which is less likely to cause symptoms prompting patients to seek care. The resource challenges and delays of in-person care caused by the COVID-19 pandemic likely accounted for an observed decrease in the incidence of reported CT cases during 2020 compared to 2019 [1]. In contrast, reported NG and syphilis rates were increased in 2020. As non-pandemic-related preventive services are restored in health departments and other clinical settings, there are opportunities to establish improved mechanisms for STI education, testing, and retesting to prevent initial and recurrent STIs. Our findings did not favor one retesting time interval over another and, consequently, do not contradict current national guidelines to retest 3 months after treatment. We recommend retesting at 3 months after the initial infection and treatment with considerations for earlier retesting (i.e., as early as 2 months) depending on unique needs and availability of military personnel.
